# Metabolic Risk Factors Associated With Chronic Kidney Disease in a Middle-Aged and Elderly Taiwanese Population: A Cross-Sectional Study

**DOI:** 10.3389/fmed.2021.748037

**Published:** 2021-11-16

**Authors:** Mei-Chun Lu, I-Ju Chen, Le-Tien Hsu, Ying-Jen Chen, Meng-Ting Tsou, Tao-Hsin Tung, Jau-Yuan Chen

**Affiliations:** ^1^Department of Family Medicine, Chang Gung Memorial Hospital, Linkou Branch, Taoyuan City, Taiwan; ^2^Department of Gynecology and Obstetrics, Chang Gung Memorial Hospital, Taoyuan City, Taiwan; ^3^Division of General Internal Medicine and Geriatrics, Department of Internal Medicine, Chang Gung Memorial Hospital, Taoyuan City, Taiwan; ^4^Department of Family Medicine and Occupation Medicine, MacKay Memorial Hospital, Taipei City, Taiwan; ^5^Department of Nursing, and Management, MacKay Junior College of Medicine, Taipei City, Taiwan; ^6^Evidence-Based Medicine Center, Taizhou Hospital of Zhejiang Province Affiliated to Wenzhou Medical University, Zhejiang, China; ^7^College of Medicine, Chang Gung University, Taoyuan City, Taiwan

**Keywords:** chronic kidney disease, prevalence, middle-aged and elderly, metabolic risk factors, metabolic syndrome

## Abstract

**Background:** This study aimed to quantify the proportion of participants with chronic kidney disease (CKD) and associated metabolic risk factors in a middle-aged and elderly population in Guishan District, Taoyuan City, Taiwan.

**Methods:** This cross-sectional study enrolled residents aged 50–90 years living in one community. All participants received a standardized personal interview, including a structured questionnaire, anthropometric measurements, and blood samples collected for laboratory testing. CKD was defined as the presence of kidney damage (urine albumin-creatinine ratio ≥30 mg/g) or estimated glomerular filtration rate (eGFR) < 60 mL/min/1.73 m^2^. Multiple logistic regression models were used to evaluate the risk factors associated with CKD.

**Results:** A total of 400 participants were enrolled. The overall proportion of participants with CKD was 20.5% (95% confidence interval [CI]: 16.54–24.46%). The proportions of participants with CKD among those aged 50–64, 65–74, and 75 years and over were 17.7, 18.8, and 35.7%, respectively (*p* = 0.01). Multiple logistic regression model revealed that elevated blood pressure (odds ratio [OR] = 2.23, 95% CI: 1.16–4.30), hyperglycemia (OR = 2.87, 95% CI: 1.64–5.00), hyperuricemia (OR = 1.38, 95% CI: 1.14–1.69), and metabolic syndrome (OR = 2.30, 95% CI: 1.31–4.06) were significantly associated with CKD.

**Conclusions:** The prevalence of CKD in the study population was high. Hypertension, hyperglycemia, hyperuricemia, and metabolic syndrome are significantly associated with CKD in a middle-aged and elderly population in Taiwan.

## Introduction

Taiwan has had the highest prevalence of end-stage renal disease (ESRD) worldwide for more than a decade (1). Besides in Taiwan, the increase in ESRD populations is also of great concern for the United States because ESRD expenditures are gradually consuming greater proportions of the healthcare budget (2). The prevalence of chronic kidney disease (CKD) is higher in older adults, reaching 37.2% in older patients (aged ≥ 65 years), while the prevalence of CKD is 11.93% in adults of all ages (age ≥ 20 years) in Taiwan (3). Treatments for CKD have the greatest effect on slowing the rate of disease progression when started early. CKD can be divided into five stages based on the appearance of kidney damage or estimated glomerular filtration rate (eGFR) < 60 mL/min/1.73 m^2^. Unfortunately, most patients are unaware of their disorders until they are in later stages (3).

Metabolic syndrome is another global health concern with a rising prevalence. It is defined as a cluster of disorders and risk factors for cardiovascular disease, including abdominal obesity, hyperglycemia (including impaired glucose tolerance and diagnosed diabetes), dyslipidemia, and elevated blood pressure. Many studies have demonstrated a strong association between metabolic syndrome or metabolic components and the risk for subsequent development of type 2 diabetes (4–8), cardiovascular disease (9–13), hyperuricemia, gout (14–16), and CKD (17–21). These factors interact as both cause and effect, and the multiple mechanisms involved in the development of CKD in patients with metabolic syndrome have not been well-established.

This study aimed to quantify the proportion of participants with CKD and associated risk factors, especially metabolic syndrome, in a middle-aged and elderly population in Taiwan. Understanding the proportion of subjects with CKD and important risk factors may lead to early detection of CKD and prevention of ESRD, cardiovascular disease, and reduce associated mortality.

## Materials and Methods

### Study Design and Participants

This community-based, cross-sectional study enrolled 400 volunteer residents aged 50 years and over who lived in Guishan District, Taoyuan City, Taiwan, between January 2014 and October 2014. We recruited these volunteers at gatherings in towns such as temples and community centers. Each participant received a standardized personal interview, including a structured questionnaire, anthropometric measurements, and collection of blood samples, on a single day.

### Data Collection

Data were collected from participants using a structured questionnaire requesting information on smoking habits, physical exercise habits, medical history, and current medications. Height and weight were measured to the nearest 0.1 kg and 0.1 cm using an automatic scale. Body mass index (BMI) was calculated as the ratio of weight to height in meters squared (kg/m^2^). Waist circumference was measured at the midpoint between the lower border of the rib cage and the upper iliac crest on the mid-axillary line. Blood pressure was determined using an automatic sphygmomanometer on the right upper arm after at least 15 min of rest. Venous blood samples were collected after overnight fasting for at least 12 h. All blood samples were stored in a refrigerator at 4°C and analyzed at the clinical laboratory of Linkou Chang Gung Memorial Hospital, which was certified by the College of American Pathologists. Serum and urine creatinine levels were measured using the isotope dilution mass spectrometry (IDMS) traceable colorimetric method. Urinary albumin levels were measured using a turbidimetric immunoassay. Urine specimens were obtained in the morning and scheduled to avoid contamination by menstrual blood. The study protocol was approved by Chang-Gung Medical Foundation Institutional Review Board (102-2304B), and all participants provided signed informed consent before enrollment.

### Definitions of Measurement Cutoffs and Calculations

Chronic kidney disease (CKD) is defined as the presence of kidney damage (urine albumin-creatinine ratio ≥ 30 mg/g) or decreased renal function with an eGFR < 60 mL/min/1.73 m^2^. The eGFR was calculated using the 2009 Chronic Kidney Disease Epidemiology Collaboration (CKD-EPI) equation, as suggested by the Kidney Disease: Improving Global Outcomes 2012 clinical practice guideline.

2009 CKD-EPI equation:

eGFR = 141 × min (Scr /κ, 1)^α^ × max(Scr /κ, 1)^−1.209^ × 0.993^Age^ × 1.018 [if female] × 1.159 [if black] Scr is serum creatinine in mg/dL, κ is 0.7 for females and 0.9 for males, α is −0.329 for females and −0.411 for males, min indicates the minimum Scr/κ or 1, max indicates the maximum Scr/κ or 1 (22).

Metabolic syndrome was diagnosed when a participant had at least three of the following five medical conditions, as described by The Third Report of the National Cholesterol Education Program Expert Panel on Adult Treatment Panel Asian diagnostic criteria: (1) elevated blood pressure (systolic blood pressure ≥ 130 mm Hg or diastolic blood pressure ≥ 85 mm Hg, or drug treatment of previously diagnosed hypertension); (2) hyperglycemia (fasting plasma glucose ≥ 100 mg/dL, or established diagnosis of diabetes) (3) hypertriglyceridemia (serum triglyceride ≥ 150 mg/dL, or drug treatment of hypertriglyceridemia); (4) low high-density lipoprotein cholesterol (HDL-C) level (<40 mg/dL for men and <50 mg/dL for women); and (5) central obesity (≥90 cm for males and ≥80 cm for females).

BMI categories were defined as follows: (1) normal weight: BMI < 23 kg/m^2^; (2) overweight: BMI of 23 to <25 kg/m^2^; and (3) obesity: BMI ≥25 kg/m^2^, according to ranges established for Asian populations (23).

### Statistical Analysis

Data are presented as mean ± standard deviation (SD) for continuous variables and number of participants (%) for categorical variables. Differences in the mean values of continuous variables were examined using an independent *t*-test and one-way analysis of variance. The chi-squared test and χ^2^-trend test were used for differences in proportions between categorical variables. The Mantel–Haenszel χ^2^ test was used to analyze stratified categorical data. Multiple logistic regression models were developed to investigate the association of five metabolic components of metabolic syndrome and CKD. Traditional factors known to be associated with metabolic factors and CKD were adjusted. We also selected the factors that showed significant differences in CKD and non-CKD participants in the current study as covariates in the regression models. All statistical analyses were performed using SPSS for Windows, SPSS version 27.0.1.0 (SPSS Inc., Chicago, IL). A *p*-value of <0.05 was considered significant.

## Results

### Proportions of Participants With Chronic Kidney Disease

**Figure 1:**
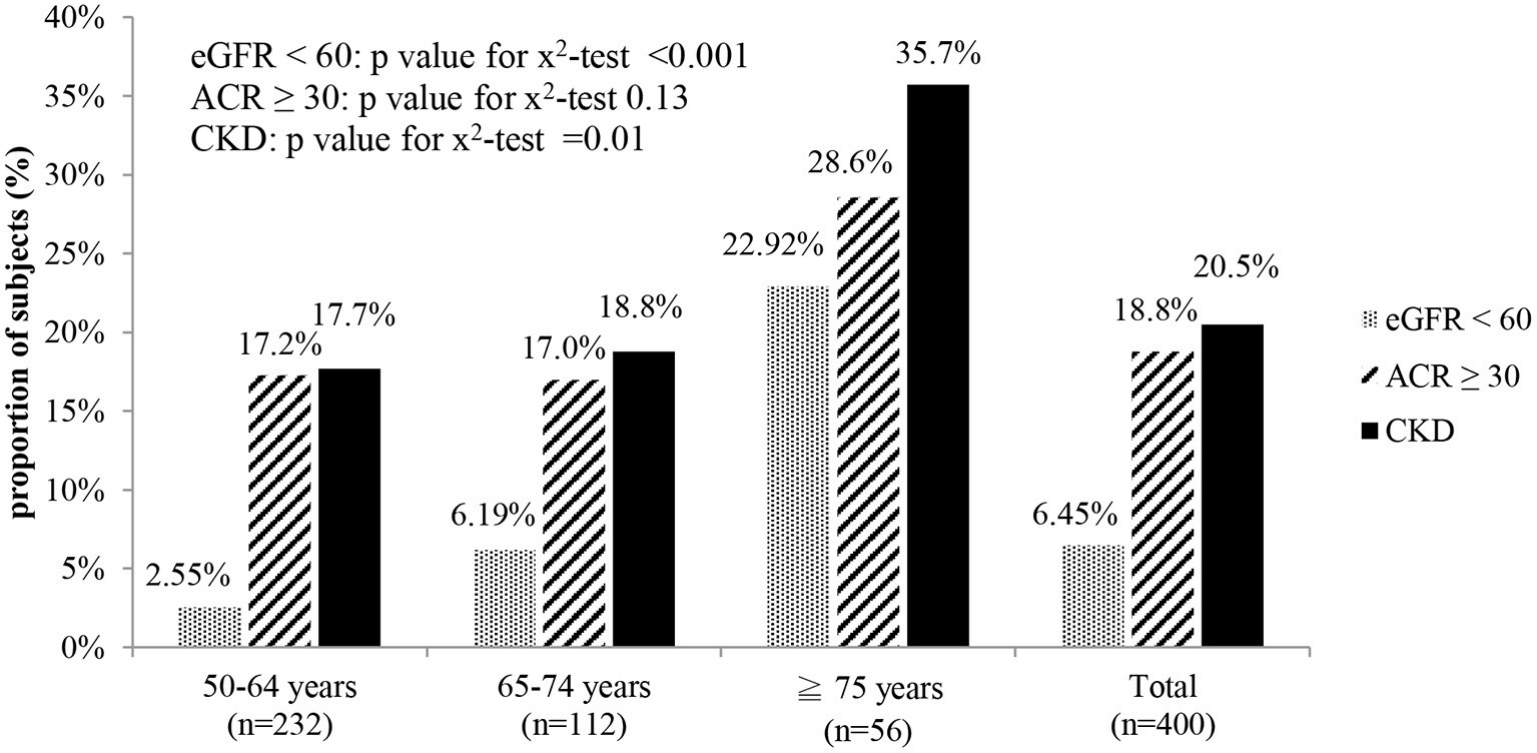
Proportion of subjects with CKD in different age groups. eGFR < 60: estimated glomerular filtration rate < 60 mL/min/1.73 m^2^. ACR ≥ 30: urine albumin creatinine ratio ≥ 30 mg/g. CKD: eGFR < 60 or ACR ≥ 30.

As shown in Figure 1, the overall proportion of participants with CKD in the study population (age ≥ 50 years) was 20.5% (95% confidence interval [CI]: 16.54–24.46%). The oldest group had the highest proportion of participants with CKD, whether diagnosed by microalbuminuria, declined eGFR, or either one of them. More than one in three participants older than 75 years old (proportion: 35.7%, 95% CI: 23.16–48.26%) were diagnosed with CKD in this community-based study.

### Clinical Characteristics of the Study Population

A total of 400 participants were categorized into three different age groups: 50–64 years old (*n* = 232), 65–74 years old (*n* = 112), and ≥75 years (*n* = 56). The ratio of waist circumference to height (WC/height), diastolic blood pressure, total cholesterol, low-density lipoprotein cholesterol (LDL-C), alanine aminotransferase (ALT), creatinine, and eGFR were significantly different between the age subgroups (Table 1). We also compared demographic, anthropometric, and clinical characteristics between the CKD and the non-CKD groups and found that age, waist circumference, the ratio of waist circumference to height, systolic blood pressure, diastolic blood pressure, fasting glucose, triglyceride, HDL-C, LDL-C, the ratio of triglyceride to HDL-C, and uric acid were the factors significantly associated with CKD (Table 1).

**Table 1:** Demographic, anthropometric, and biochemical characteristics of screened subjects with and without chronic kidney disease.

**Variables**		**Age**				**Chronic kidney disease**		
	**Total**	**50–64**	**65–74**	**≧75**	***p*-value**	**Yes**	**No**	***p*-value**
	**(*n* = 400)**	**(*n* = 232)**	**(*n* = 112)**	**(*n* = 56)**		**(*n* = 81)**	**(*n* = 319)**	
	**Mean ± SD**	**Mean ± SD**	**Mean ± SD**	**Mean ± SD**		**Mean ± SD**	**Mean ± SD**	
Age (year)	64.47 ± 8.45	58.53 ± 4.01	69.31 ± 2.89	79.34 ± 3.42	<0.001	66.67 ± 9.71	63.91 ± 8.02	0.02
BMI (kg/m^2^)	24.55 ± 3.57	24.54 ± 3.66	24.76 ± 3.26	24.16 ± 3.78	0.59	25.10 ± 3.93	24.41 ± 3.46	0.12
WC (cm)	85.07 ± 9.68	84.30 ± 9.76	85.68 ± 8.59	87.02 ± 11.14	0.12	87.11 ± 10.63	84.55 ± 9.37	0.03
WC/height	0.54 ± 0.06	0.53 ± 0.06	0.54 ± 0.05	0.55 ± 0.07	0.01	0.55 ± 0.06	0.53 ± 0.06	0.02
SBP (mmHg)	129.5 ± 16.71	128.00 ± 16.65	130.80 ± 15.66	133.13 ± 18.47	0.07	135.38 ± 16.51	128.01 ± 16.46	<0.001
DBP (mmHg)	76.93 ± 11.36	78.75 ± 11.33	76.80 ± 10.55	69.66 ± 10.26	<0.001	79.99 ± 13.42	76.15 ± 10.66	0.01
FPG (mg/dL)	96.23 ± 25.73	94.72 ± 20.67	98.00 ± 26.51	98.96 ± 39.59	0.38	105.31 ± 40.20	93.93 ± 19.95	0.02
Triglyceride (mg/dL)	122.07 ± 65.97	122.55 ± 59.99	123.79 ± 74.09	116.68 ± 73.08	0.80	145.95 ± 87.39	116.01 ± 57.94	0.004
Total cholesterol (mg/dL)	197.15 ± 35.71	200.98 ± 36.98	194.17 ± 32.93	187.20 ± 33.70	0.02	190.72 ± 34.94	198.78 ± 35.77	0.07
HDL-C (mg/dL)	54.43 ± 13.93	54.75 ± 13.91	54.52 ± 13.81	52.93 ± 14.44	0.68	51.04 ± 15.16	55.29 ± 13.49	0.01
LDL-C (mg/dL)	118.37 ± 32.11	121.75 ± 34.41	114.96 ± 27.87	111.20 ± 28.57	0.04	110.68 ± 29.16	120.32 ± 32.57	0.02
TG/HDL-C	2.55 ± 1.96	2.54 ± 1.81	2.57 ± 2.04	2.59 ± 2.42	0.97	3.37 ± 2.72	2.35 ± 1.66	<0.001
Uric acid (mg/dL)	5.75 ± 1.41	5.75 ± 1.43	5.70 ± 1.40	5.82 ± 1.39	0.87	6.27 ± 1.77	5.62 ± 1.28	0.002
ALT (U/L)	22.63 ± 12.95	24.33 ± 13.26	20.61 ± 13.12	19.59 ± 10.04	0.01	24.11 ± 14.51	22.25 ± 12.52	0.25
HS-CRP (mg/dL)	2.79 ± 6.00	2.94 ± 5.91	2.16 ± 5.15	3.41 ± 7.72	0.37	4.23 ± 8.99	2.42 ± 4.92	0.08
Creatinine (mg/dL)	0.78 ± 0.43	0.71 ± 0.21	0.81 ± 0.47	0.99 ± 0.78	<0.001	1.06 ± 0.83	0.7 ± 0.17	<0.001
eGFR (ml/min/1.73 m^2^)	89.66 ± 17.82	95.85 ± 13.07	85.54 ± 17.68	72.23 ± 21.19	<0.001	76.14 ± 28.79	93.14 ± 11.33	<0.001
ACR ≧ 30 mg/g (*n*, %)	75, 18.75%	40, 17.24%	19, 16.96%	16, 28.57%	0.13	75, 91.46%	0, 0.00%	<0.001
Smoking (*n*, %)	43, 10.75%	29, 12.50%	8, 7.14%	6, 10.71%	0.32	35, 10.97%	8, 9.88%	0.78
Physical exercise (*n*, %)	328, 82.00%	182, 78.45%	97, 86.61%	49, 87.50%	0.09	259, 81.19%	69, 85.19%	0.40

*Clinical characteristics are expressed as mean ±SD for continuous variables. P-values were derived from one-way analysis of variance (one-way ANOVA) and independent two-sample t-test for continuous variables, and Chi-squared test for categorical variables*.

*BMI, body mass index; WC, Waist circumference; SBP, systolic blood pressure; DBP, diastolic blood pressure; FPG, fasting plasma glucose; HDL-C, high-density lipoprotein cholesterol; LDL-C, low-density lipoprotein cholesterol; TG, triglyceride; ALT, alanine aminotransferase; HS-CRP, high sensitivity C-reactive protein; eGFR, estimated glomerular filtration rate; ACR, urine albumin creatinine ratio*.

### Associations Between Metabolic Syndrome and Chronic Kidney Disease

Table 2 shows the associations between the five components of metabolic syndrome and CKD. Elevated blood pressure, hyperglycemia, hypertriglyceridemia, and low HDL-C were significantly associated with an increased age-specific prevalence of CKD, while central obesity showed a similar trend but without statistical significance.

**Table 2:** Associations between metabolic risk factors and chronic kidney disease in subjects by age group.

	**50–64 years**	**65–74 years**	****≧**75 years**	**Total**	***p*-value**
	**CKD**	**CKD**	**CKD**	**CKD**	
	**Prevalence(95% CI)**	**Prevalence(95% CI)**	**Prevalence(95% CI)**	**Prevalence(95% CI)**	
Metabolic syndrome	24.7 (17.6–31.7)	35.0 (27.2–42.8)	46.2 (38.0–54.3)	31.5 (23.9–39.1)	<0.001
Metabolic components
Elevated blood pressure^a^	21.6 (16.6–26.7)	26.9 (21.5–32.4)	39.5 (33.5–45.5)	26.3 (20.9–31.7)	<0.001
Central obesity^b^	19.2 (14.1–24.3)	21.4 (16.1–26.7)	37.1 (30.9–43.4)	22.6 (17.2–28.0)	0.34
Hyperglycemia^c^	32.2 (23.7–40.7)	28.9 (20.7–37.2)	57.9 (48.9–66.9)	35.3 (26.6–44.0)	<0.001
Hypertriglyceridemia^d^	24.3 (16.7–32.0)	31.0 (22.8–39.3)	50.0 (41.1–58.9)	29.8 (21.7–37.9)	0.003
Low HDL-C^e^	24.2 (16.2–32.2)	36.7 (27.6–45.7)	47.1 (37.7–56.4)	31.2 (22.5–39.9)	0.002

a *SBP ≧ 130 mmHg or DBP ≧ 85 mmHg or self-reported hypertension*.

b *Waist circumference ≥90 cm in men or ≥80 cm in women*.

c *Fasting blood glucose ≥ 100 mg/dL or self-reported diabetes mellitus*.

d *TG ≥ 150 mg/dL*.

e *HDL-C < 40 mg/dL in men or <50 mg/dL in women*.

*HDL-C, high-density lipoprotein cholesterol; TG, triglyceride; CKD, chronic kidney disease; CI, confidence interval*.

The effects of metabolic syndrome on CKD were further examined using multiple logistic regression models (Table 3). Traditional factors known to be associated with metabolic factors and CKD were selected in the models, such as age, sex, and BMI categories. Additional factors showing significant differences in the CKD group in the preliminary analysis (Table 1) were also selected in the models, such as uric acid. After adjusting for age, sex, BMI categories, and uric acid, metabolic syndrome (yes vs. no, odds ratio [OR] = 2.30, 95% CI: 1.31–4.06) were independently significantly associated with CKD (Table 3 model A). Another model was used to examine each of the five components of metabolic syndrome. After adjusting for the above-mentioned confounding factors, elevated blood pressure (yes vs. no, OR = 2.23, 95% CI: 1.16–4.30) and hyperglycemia (yes vs. no, OR = 2.87, 95% CI: 1.64–5.00) were still the metabolic components significantly associated with CKD (Table 3 model B).

**Table 3:** Multiple logistic regressions: the effects of metabolic syndrome and metabolic components on the chronic kidney disease among screened population.

**Variables**	**Odds ratio**	**CKD vs. non-CKD 95% CI**	***p* value**
**Model A (metabolic syndrome)**			
Age			
(65–74 vs. 50–64)	1.07	(0.58–1.95)	0.83
(≧75 vs. 50–64)	2.45	(1.24–4.84)	0.01
Sex (men vs. women)	0.84	(0.48–1.49)	0.56
BMI			
(Overweight vs. normal)	1.01	(0.54–1.91)	0.97
(Obese vs. normal)	0.94	(0.47–1.87)	0.86
Metabolic syndrome (yes vs. no)	2.30	(1.31–4.06)	0.004
Uric Acid (mg/dL)	1.39	(1.14–1.68)	0.001
**Model B (metabolic components)**			
Age			
(65–74 vs. 50–64)	1.00	(0.53–1.88)	0.99
(≧75 vs. 50–64)	2.44	(1.21–4.93)	0.01
Sex (men vs. women)	0.76	(0.42–1.38)	0.37
BMI			
(Overweight vs. normal)	1.26	(0.63–2.51)	0.52
(Obese vs. normal)	1.09	(0.49–2.45)	0.84
Elevated blood pressure (yes vs. no)	2.23	(1.16–4.30)	0.02
Central obesity (yes vs. no)	0.71	(0.35–1.44)	0.34
Hyperglycemia (yes vs. no)	2.87	(1.64–5.00)	<0.001
Hypertriglyceridemia (yes vs. no)	1.25	(0.69–2.26)	0.46
Low HDL-C (yes vs. no)	1.61	(0.88–2.93)	0.12
Uric Acid (mg/dL)	1.38	(1.14–1.69)	0.001

*Goodness-of-fit test: The p-value of the Hosmer–Lemeshow test is 0.74 and 0.16 for logistic regression model A and model B, respectively*.

## Discussion

In our study, hypertension, hyperglycemia, hyperuricemia, and metabolic syndrome were significantly associated with CKD in a middle-aged and older population in Taiwan. In several studies among different countries and races, metabolic syndrome has been identified as a risk factor for developing CKD (24–28). In Japan, Tozawa et al. (29) followed up 6,371 people without CKD or diabetes mellitus for 5 years and found that the relative risk of developing CKD was 1.86 (95% CI: 1.43–2.41, *p* < 0.0001) in those with metabolic syndrome after adjusting for age, sex, current cigarette smoking, and alcohol drinking habits. In the United States, Kurella et al. (30) enrolled 10,096 nondiabetic participants with 9 years of follow-up, likewise revealing that metabolic syndrome was independently associated with an increased risk for incident CKD in nondiabetic adults; the OR of incident CKD among participants with metabolic syndrome was 1.24 (95% CI, 1.01–1.51) after adjusting for the subsequent development of diabetes and hypertension. In the present study, metabolic syndrome also serves as an independent risk factor for the development of CKD (yes vs. no, OR = 2.43, 95% CI: 1.38–4.29) in a middle-aged and elderly population in Taiwan after adjusting for age, sex, BMI categories, and uric acid.

Each component of metabolic syndrome can cause renal damage; however, the components may not contribute equally to the risk of developing CKD (25, 31, 32). Many studies have further reported the gradient associations between CKD risk and the number of components of metabolic syndrome (29, 30, 32–34). The multiple mechanisms of renal damage caused by each metabolic syndrome component and their interactions with each other are not yet thoroughly understood. In the present study, elevated blood pressure and hyperglycemia were independent risk factors for CKD, while other components did not reach statistical significance after adjusting for confounding factors.

High-normal blood pressure is significantly associated with microalbuminuria when compared with optimal blood pressure, and the increase in urinary protein causes injury to tubular cells, leading to interstitial inflammation and fibrosis (35, 36). Previous studies have also revealed that elevated blood pressure, as a component of metabolic syndrome, is an independent risk factor for CKD development. Cao et al. (37) enrolled 11,274 participants and found that CKD risk was significantly greater (OR, 1.30; 95% CI: 1.03–1.63) in men with high-normal blood pressure than in those with optimal blood pressure. Song et al. (32) followed 75,468 urban workers for 2 years and found that the OR of metabolic syndrome-related to reduced eGFR was 1.43 (95% CI, 1.13–1.83). In addition, lower blood pressure targets (130/80 mmHg) are strongly associated with better renal outcomes (38). Thus, aggressive blood pressure control is suggested for the management of patients with metabolic syndrome and mild renal function decline to promote a better prognosis.

Hyperglycemia, including previously diagnosed diabetes and impaired fasting glucose, is another component of metabolic syndrome that is significantly associated with CKD in the present study. Increased glomerular filtration rate, also called hyperfiltration, is a proposed mechanism for renal injury in diabetes, which has been hypothesized to cause intra-glomerular hypertension leading to albuminuria and reduced glomerular filtration rate. Hyperfiltration also occurs in patients with impaired fasting glucose and can be used as a predictor of diabetic nephropathy (39–42).

Hypertriglyceridemia, low HDL-C levels, and central obesity were not significantly associated with CKD in the present study. Several other studies have reported similar results. Although metabolic syndrome itself is an independent risk factor associated with CKD, dyslipidemia (including both hypertriglyceridemia and low HDL-C level) is not significantly associated with the development of CKD (26, 29, 30, 33, 34, 43). Some studies have shown that hypertriglyceridemia or low HDL-C levels are only significantly associated with the development of CKD in patients with metabolic syndrome (32, 44). Regarding central obesity, a longitudinal study in China found that people with both central and peripheral obesity had higher risks of elevated urine albumin-creatinine ratio, even after adjusting for multiple factors (OR: 1.14, 95% CI: 1.07–1.21, *p* < 0.001) (45). However, there is no consensus on the role of central obesity in the development of CKD (33, 35, 44, 46, 47). Differences in race, large disparities in participants' ages, the definitions of CKD, and adjusted confounding factors in these studies might be other reasons for discrepancies between studies.

CKD and metabolic syndrome are both considered inflammatory diseases (48, 49). As a downstream marker of inflammation, C-reactive protein is widely used to represent inflammatory conditions. It has been associated with CKD in previous studies (50, 51). However, in our present study, although the mean high sensitive C-reactive protein level was higher in the CKD group, the difference was not statistically significant (Table 1). Further follow-up studies are needed to confirm this association.

The present study has several limitations. First, this was a cross-sectional study; thus, the causal relationship between CKD and the associated risk factors could not be evaluated and determined. Second, the number of participants in this study was relatively small, and all participants were recruited from only a single community. Third, volunteer bias may exist because participation is on a volunteer basis. Volunteer bias is defined as the bias that comes from the fact that a particular sample contains only those participants who volunteer to participate in the study. Those who participate and find the topic particularly interesting are more likely to volunteer, and the same is true of those who are expected to be evaluated on a positive level (52). In addition, we recruited volunteer participants to join our study at gatherings in towns such as temples and community centers. This means that people who require considerable assistance or frequent medical care have a lower possibility of being recruited. Volunteer bias could potentially influence the prevalence of CKD and the associated risk factors, and the association between CKD and associated risk factors in those who require substantial assistance or care might not be observed in the present study. Fourth, it was difficult to collect all the participants' first morning voids in this community-based study. This could lead to biased estimation for some participants' urine albumin-creatinine ratios. Finally, insulin resistance is a known risk factor for CKD, but this has not been discussed in this study, further studies are needed.

## Conclusions

CKD is found to be significantly associated with older age, elevated uric acid level, and metabolic syndrome after adjusting for sex and BMI categories in a middle-aged and elderly population in Taiwan. Among the components of metabolic syndrome, elevated blood pressure and hyperglycemia are independently associated with the risk of CKD. For patients with metabolic syndrome, clinical interventions such as lifestyle modification, weight reduction, the use of medications to correct elevated blood pressure, hyperglycemia, dyslipidemia, and hyperuricemia may prevent or delay the progression of CKD.

## Data Availability Statement

The original contributions presented in the study are included in the article/supplementary material, further inquiries can be directed to the corresponding author/s.

## Ethics Statement

The studies involving human participants were reviewed and approved by Chang-Gung Medical Foundation Institutional Review Board. The patients/participants provided their written informed consent to participate in this study. Written informed consent was obtained from the individual(s) for the publication of any potentially identifiable images or data included in this article.

## Author Contributions

Conceived and supervised the study: J-YC. Designed the study: T-HT and J-YC. Performed the study: M-CL and J-YC. Analyzed the data and interpreted the results: M-CL, I-JC, L-TH, Y-JC, M-TT, T-HT, and J-YC. Writing-original draft: M-CL. Writing-editing: M-CL, I-JC, L-TH, T-HT, and J-YC. All authors contributed to the article and approved the submitted version.

## Funding

This study was supported by Chang Gung Memorial Hospital (grants CORPG3C0171~3C0172, CZRPG3C0053, CORPG3G0021, CORPG3G0022, and CORPG3G0023).

## Conflict of Interest

The authors declare that the research was conducted in the absence of any commercial or financial relationships that could be construed as a potential conflict of interest.

## Publisher's Note

All claims expressed in this article are solely those of the authors and do not necessarily represent those of their affiliated organizations, or those of the publisher, the editors and the reviewers. Any product that may be evaluated in this article, or claim that may be made by its manufacturer, is not guaranteed or endorsed by the publisher.
